# Economic evaluation of task-shifting approaches to the dispensing of anti-retroviral therapy

**DOI:** 10.1186/1478-4491-10-32

**Published:** 2012-09-13

**Authors:** Nicola Foster, Diane McIntyre

**Affiliations:** 1 Health Economics Unit, School of Public Health and Family Medicine, University of Cape Town, Observatory, Cape Town, South Africa

**Keywords:** Task-shifting, Pharmaceutical care models, Skills mix, Anti-retroviral therapy

## Abstract

**Background:**

A scarcity of human resources for health has been identified as one of the primary constraints to the scale-up of the provision of Anti-Retroviral Treatment (ART). In South Africa there is a particularly severe lack of pharmacists. The study aims to compare two task-shifting approaches to the dispensing of ART: Indirectly Supervised Pharmacist’s Assistants (ISPA) and Nurse-based pharmaceutical care models against the standard of care which involves a pharmacist dispensing ART.

**Methods:**

A cross-sectional mixed methods study design was used. Patient exit interviews, time and motion studies, expert interviews and staff costs were used to conduct a costing from the societal perspective. Six facilities were sampled in the Western Cape province of South Africa, and 230 patient interviews conducted.

**Results:**

The ISPA model was found to be the least costly task-shifting pharmaceutical model. However, patients preferred receiving medication from the nurse. This related to a fear of stigma and being identified by virtue of receiving ART at the pharmacy.

**Conclusions:**

While these models are not mutually exclusive, and a variety of pharmaceutical care models will be necessary for scale up, it is useful to consider the impact of implementing these models on the provider, patient access to treatment and difficulties in implementation.

## Background

The scarcity of human resources for health (HRH) has been identified as one of the primary constraints to the provision of anti-retroviral treatment (ART) to all who need it [1-3]. Pharmaceutical services experience similar workload pressures and staff shortages as those that plague other services involving HRH. While the World Health Organization’s (WHO) guidelines recommend a minimum country average of 1 pharmacist per 2300 people, many low- and middle-income countries (LMIC) have averages far above that level. South Africa has 1 pharmacist per 4332 people [4]. In conjunction with international migration, and a preference for urban over rural posts, South Africa also experiences a strong pull of pharmacists away from the public health service and into the private sector, with approximately 24% of registered pharmacists employed in the public sector which serves more than 80% of the population [5]. This has led to a gap in the systems and processes associated with the safe provision of chronic (and specifically ART) drug treatment which is crucial as adherence is becoming of greater concern [4].

The substitution of scarce highly skilled health workers with purpose trained mid-level workers (termed task-shifting) is a logical strategy to address the scarcity of HRH [6]. The primary aim of task-shifting is to facilitate increased patient access to health services, through enabling a rapid scale-up of much needed health care interventions, and lower HRH training and salary costs [7]. In addition to the cost and efficiency benefits, mid-level workers have been found to be more likely to remain and work in rural areas and follow treatment guidelines. While quality of care has been cited as a concern by critics, studies suggest that the quality of care provided by mid-level workers is at least comparable to that of their more highly qualified colleagues [8-11]. A randomized non-inferiority trial conducted in primary health care clinics in South Africa (SA), which compared doctor- versus nurse-monitored ART care, found that nurse-monitored care was comparable to doctor-monitored ART care and, therefore, supported task-shifting [11].

The shortage of pharmacists in the public health sector of SA has led to the use of pharmacists’ assistants and nurses to support the expansion of the ART programme and to ensure that patients receive medication. However, evidence of the costs and benefits of these models is lacking. There is little consensus in the literature on the appropriate methodology to use in comparing HRH staff mix in a health system, although Fulton et al. (2011) have suggested that cost-effectiveness analyses would be useful in ensuring that appropriate comparisons are made [7]. Thus far, the focus of staff mix evaluations has been on the delegation of tasks from doctors to physician assistants [9,12-18], from doctors to nurses [11,19-24], and from nurses to community health workers [25-30], with a paucity of studies referring to pharmaceutical care staff [31]. This study, therefore, aims to evaluate critically the indirectly supervised pharmacists’ assistant (ISPA) and nurse-based pharmaceutical care models against the standard of care which involves a pharmacist dispensing ART, on the basis of cost, waiting and travel time and patient preference.

## Methods

### Study design and setting

A cross-sectional, mixed method study design was used. Data were collected using patient exit interviews, time and motion studies, and expert interviews. The study was conducted in a peri-urban district of the Western Cape province of SA.

### Sampling

Within the district, health care facilities were grouped based on the pharmaceutical care model they used, with two facilities sampled per group. For each group an equal number of respondents was sampled, and the desired sample size per facility was proportional to the number of patients on treatment at the facility. Systematic sampling was used to select adult respondents on ART to interview while waiting for medication.

### Data collection

A questionnaire was developed, piloted and interviewer-administered, to collect data regarding the direct and indirect costs incurred in accessing treatment as well as the acceptability of the service provided. Direct costs included the cost of transport, any facility fees incurred, the cost of employing someone to take over tasks (such as child minding), accommodation if sleeping over, and the cost of food and telecommunication while waiting at the facility. Indirect costs were estimated by asking respondents about income lost from taking time to come to the facility, as well as the cost of time spent travelling to the facility. Patient waiting time was estimated by attaching a printed form to each patient’s folder, noting the time at which they entered the facility and pharmacy or nurse dispensers were asked to note the time at which respondents received their medicine. Collecting medication was considered to be the last contact point in the service chain. The difference in time was aggregated per facility and added to the average travel time to estimate the indirect cost per respondent per visit.

Health service expenditure and staffing data were obtained through facility observation and expert interviews with service managers. In order to determine the time spent by nurses on dispensing-related activities, the researcher observed practice and asked nurses to estimate time spent dispensing if a total of 100% represents one work day. The cost of HRH was estimated from Department of Health (DOH) advertisements for posts and discussed with service managers to ensure accuracy.

The potential cost of upgrading a medicine room to a dispensary, as required by the Pharmacy Act for a pharmacist’s assistant to work in, was determined from a case study of a facility within the same district where the research was conducted, and sourced from the non-governmental organization (NGO) which paid for the upgrade.

Respondent preferences were explored by asking whether they would prefer collecting medication from a local clinic or hospital, in addition to asking whether a nurse or pharmacist/pharmacist’s assistant dispensing was preferable. Respondents were also asked to explain their preferences.

### Data analysis

Statistical analysis of data was conducted using STATA 10® [32]. For examining health care costs, Microsoft Excel® was used. All costs are presented in 2009/2010 prices and estimated costs were converted to US dollars at the average exchange rate of the US dollar to South African rand for the 2010 financial year of US$1 = R7.80 [33].

The cost analysis was conducted from a societal perspective, and includes health service and patient costs. Given that the study is comparative, costs that are common across pharmaceutical care models were excluded [34]. For example, given that the cost of ARV drugs per patient does not vary between models, these costs were excluded [35]. The costs of staff employed by donors were included, in line with the donor mandate that these functions are to be taken over by the DOH in the future [36]. Although the inclusion of time costs is debated in the literature [37-39], patients’ waiting and transport time, which differed significantly between facilities and could be seen as a measure of efficiency of service provision, were taken into account and the opportunity cost was calculated using the minimum hourly rate for a domestic worker in South Africa, US$0.94 per hour [40]. While cost analysis assumes that outcomes are similar, an element of outcome measurement was included by considering the acceptability of the respective services. The open-ended or discussion questions of the exit interview responses were analyzed using domain analysis during which topics were identified, assigned a code, and domains and sub-categories explored. A list of codes was generated and used to explore the common (and different) perspectives of the respondents [41].

Preliminary results of the analysis were discussed with service managers to confirm assumptions and validate results.

### Ethics

The research was approved by the University of Cape Town Health Science Faculty Human Research Ethics Committee as well as by the Western Cape Department of Health. The study was conducted in adherence to the Declaration of Helsinki of the 25^th^ World Medical Assembly and all respondents participated on the basis of written informed consent [42].

## Results

Only 19 patients refused to be interviewed. Of the 230 patient exit interviews conducted, 6 were excluded from the analysis as respondents were either not on ART yet or were collecting medication for someone else.

### Respondent characteristics

The characteristics of respondents are summarized in Table 1.

**Table 1:** Profile of respondents

**Characteristics**	**Number (*n*** **=** ***224*)**	**%**
Gender		
male	62	27.7%
female	162	72.3%
Age		
mean age ± standard deviation	36.2 ± 9.2 years	
Employment		
unemployed	140	62.8%
employed full-time	41	18.4%
employed part-time	42	18.8%

The unequal gender distribution of people who access ART in public health facilities has been well documented [43-45]. The age distribution of respondents, with a mean age of 36 years, is in line with the national profile of those on ART [46]. The unemployment rate is significantly higher at 62.8% than the overall Western Cape provincial estimate of 20.3% of the population [47]. This can partly be attributed to the organization of the SA health system whereby those employed are more likely to use private health care paid for by medical insurance.

### Facility characteristics

The study was conducted in a peri-urban district, which includes some more rural facilities. Table 2 summarizes some of the key aspects of the service provided by the facilities sampled.

**Table 2:** Summary characteristics of ART service by facility

	**Group A: full-time pharmacist**		**Group B: pharmacist assistant under indirect supervision**		**Group C: nurse-driven**	
	**Pharm clinic 1**	**Pharm clinic 2**	**ISPA clinic 1**	**ISPA clinic 2**	**Nurse clinic 1**	**Nurse clinic 2**
Level of service	CDC^a^	clinic	CDC	CDC	clinic	CDC
Pharmaceutical care delivery system	A full-time pharmacist and pharmacist’s assistant dispenses medication on request, and sends the patient-ready packs to nurses to dispense to patients.	Full-time pharmacist dispenses directly to patients, gives adherence and side-effects counselling.	PA working under indirect supervision dispenses directly to patients from the general pharmacy.	PA working under indirect supervision dispenses directly to patients from the general pharmacy.	Nurse does the clinical examination and dispenses ART from patient-ready packs ordered from the central pharmacy.	Nurse does the clinical examination and dispenses ART from patient-ready packs ordered from the central pharmacy.
Staff assisting in pharmaceutical care related	1 Pharm	0.66 Pharm	0.2 SPharm^b^	0.2 SPharm	0.2 SPharm	0.2 SPharm
	1 PA	0.66 PA	1 PA	1 PA	0.2 PA	0.2 PA
	0.6 Nurse	0.66 PA		0.6 Nurse	0.36 Nurse	0.2 Nurse
	0.6 Nurse					0.08 Nurse
Number of patients on ART	1874	829	454	621	228	210
Ratio of FTE staff to patients enrolled in care	1:586	1:419	1:378	1:345	1:300	1:309
Average number of months on ART (SD)	38 (12–52) months	28 (15–49) months	10 (4–21) months	7 (4–15) months	11 (5–15) months	11 (6–23) months

^a^Community day centre (CDC): a facility which is not open 24 hours a day, 7 days a week, but at which a broad range of primary health care services are provided. It also offers accident and emergency services but not midwifery or surgery under general anaesthesia. Also provides access to x-rays, full-time pharmacist and full-time dentist (vd Merwe, personal communication); ^b^SPharm = supervisory pharmacist who supervises a maximum of 5 pharmacists assistants. ART, anti-retroviral treatment. FTE, full time equivalent; SD, standard deviation.

Pharm clinic 1 represents a relatively well resourced health centre, and had one full-time Pharmacist (Pharm), and a Pharmacist’s Assistant (PA) exclusively dispensing ART, with two nurses using an estimated 60% of their working day in dispensing- related activities. This included taking prescriptions to the pharmacy to be filled, collecting medication and counseling patients on drug use. This arrangement came about after patients expressed their discomfort at waiting at the general pharmacy window to collect their medication. Pharm clinic 1 had the greatest proportion of Full-Time Equivalent (FTE) dispensing staff at 3.2 FTE compared to Pharm clinic 2 with 1.98 FTE. However, the ratio of patients on ART to FTE staff at Pharm clinic 1 was at 586:1 far greater than 419:1 at Pharm clinic 2.

**Figure 1:**
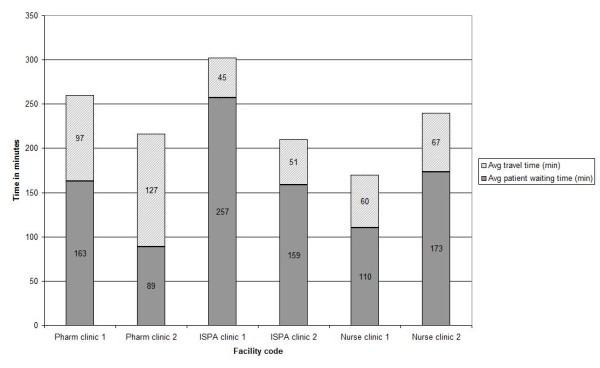
Respondent travel- and waiting-time.

The ISPA facilities had similar absolute staff FTEs (1.2 versus 1.8) and ratios of patients on ART to FTE at 378:1 and 345:1, respectively. Thus, patient load does not explain the high average waiting time at ISPA clinic 1 of four hours and seventeen minutes (see Figure 1). This facility was struggling under the lack of a fulltime ART physician, which slowed down the renewal of prescriptions and ultimately the dispensing process. ISPA clinic 2 also had significant assistance in the dispensing process by the nurse; this included the ordering of medication for patients, and ensuring that the prescription is in order for patients only coming for repeat medication.

The nurse-driven group had the fewest FTE dispensers as nurses often only provided a once or twice weekly outreach service to the site. The time spent by the PA and Supervisory Pharmacist (SPharm) at the central pharmacy in preparing prescriptions in patient-ready packs was also included in the analysis. Notably the ratio of patients on ART to FTE dispenser was considerably lower than in either of the other pharmaceutical care models at 300:1 and 308:1, respectively. One can, therefore, argue that the number of patients per FTE dispensers required is highest for the pharmacist supported model, which may be due to the increased efficiency of “learning through doing” as these ART services have been operational for longer and serve more stable patients than the newer “outreach” nurse supported facilities. It is also conceivable that staff trained in dispensing (i.e., pharmacists and pharmacist’s assistants) bring greater productive efficiency to the dispensing process and the service, therefore, takes longer to reach “saturation point” where new dispensing staff members are needed.

### Staff costs

Given that the main difference between the pharmaceutical care models relates to the cost of HRH, it was the primary focus in the cost analysis from the provider perspective. The provider (or staff) cost per patient visit (summarized in Table 3) for the nurse-driven pharmaceutical care model was at US$10.16 almost double that of the pharmacist- or ISPA- models (US$6.55 and US$5.74). This can be attributed largely to the difference in salaries between a pharmacist’s assistant and a nurse.

**Table 3:** Average cost per patient visit (*n = 244*)

	**Group A: full-time pharmacist**		**Group B: pharmacist assistant under indirect supervision**		**Group C: nurse-driven**	
	**Pharm clinic 1**	**Pharm clinic 2**	**ISPA clinic 1**	**ISPA clinic 2**	**Nurse clinic 1**	**Nurse clinic 2**
**Average provider cost**						
Staff costs	US$6.08	US$7.01	US$5.09	US$6.38	US$11.19	US$9.13
**Total cost to provider per patient visit**	**US$6.08**	**US$7.01**	**US$5.09**	**US$6.38**	**US$11.19**	**US$9.13**
**Average patient costs**						
Direct costs	US$6.20	US$3.35	US$1.59	US$1.65	US$1.14	US$3.00
Indirect costs^a^	US$5.42	US$5.09	US$7.47	US$4.15	US$3.60	US$4.84
**Total cost to patient per visit**	**US$11.62**	**US$8.45**	**US$9.06**	**US$5.81**	**U$4.74**	**US$7.84**
**Total societal costs for ART care per visit**	**US$17.70**	**US$15.46**	**US$14.16**	**US$12.19**	**US$15.93**	**US$16.97**
Percentage of respondents who incurred direct costs, and found it unaffordable (n)	90% (36)	70% (14)	77.78% (21)	68% (17)	64.29% (9)	70.97% (22)

^a^The average opportunity costs to those unemployed were estimated from the minimum daily rate for a domestic worker in South Africa (SA Department of Labor, 2010).

In the ART programme, nurses working on outreach services are generally more experienced and compensated at a higher salary level. It is, therefore, important to consider the trade-off involved in shifting dispensing related tasks from pharmacists to nurses given the scarcity of nurses. One could argue that the nurse’s time may be better spent performing clinical duties for which s/he is trained.

As would be expected, the direct costs incurred by patients accessing treatment, reflected the level of decentralization of the service. Patients paid the most at the pharmacist-led model facilities, where they spent almost four times more on transport than at the more decentralized ISPA- and nurse-driven facilities.

The cost of transport has been found to be a significant barrier to patient access to ART in other studies [48,49]. Similarly, when asked about the affordability of direct costs incurred in attending the clinic, 90% of the respondents at pharmacist-led and 67% at nurse-driven pharmaceutical care model facilities indicated that the costs incurred are unaffordable. This is certainly not surprising, given the high level of unemployment among respondents.

The average travel time is slightly less than that reported by other studies. Rosen et al. (2007) documented travel times (round trip) of between 83 and 158 minutes [50]. In this study, travel time was between 45 and 127 minutes per round trip (shown in Figure 1). However, it is interesting to note that respondents in Rosen’s study were unlikely to walk to the facility while we found that up to 97% of patients from the more decentralized sites walked to the facility. This would also impact on the transport (direct) costs reported.

From the perspective of efficiency in service delivery, it is striking that the average waiting times are on average higher in the ISPA and nurse clinics (see Figure 1) although these facilities have relatively lower numbers of ART patients per FTE staff member (see Table 2).

The costs per patient visit are summarized by the pharmaceutical care modelin Table 4. The annual cost per patient was calculated by multiplying the cost to provider and patient per visit with the average number of visits per year reported in the patient exit interviews. There was less of a difference in the societal cost than in the provider cost between the ISPA and nurse pharmaceutical care models; this is because the high cost to the provider in the nurse model is partially offset by the relatively low cost to the patient due to the decentralized nature of the service.

**Table 4:** Incremental cost of ART service use, compared between different levels of service

**Level of service**	**Average number of patient visits per year**	**Cost to provider**	**Cost to patient**	**Societal cost**	**Average annual cost per patient for baseline, incremental cost for other models**
		Average cost to provider per visit for baseline, incremental cost for other models	Average cost to patient per visit for baseline, incremental cost for other models	Average cost to society per visit for baseline, incremental cost for other models	
Full-time pharmacist (baseline)	7.78	US$ 6.55	US$ 10.04	US$16.58	US$128.99
ISPA	10.74	-US$ 0.81	-US$ 2.60	-US$3.40	US$12.56
Nurse	9.78	US$ 3.61	-US$ 3.75	-US$0.13	US$31.89

While the annual cost per patient for the pharmacist model is lower than for the ISPA model, the average cost per visit is higher. This relates to the difference in the average number of patient visits per year. Patients who are stable and adherent on treatment are given enough medication for two months and would, therefore, on average, only visit the facility six times per year. Given that the pharmacist model at more centralized facilities has been available for longer, their patients have been on treatment longer (see Table 2) and are, therefore, more likely to visit the clinic less often. This decreases the burden of care on the clinic and more patients can be seen using similar resources, while minimizing the cost to the patient.

### The cost of upgrading a medicine room to a dispensary

While the ISPA model is the cost saving option to the provider, there is an infrastructure upgrade required when moving from the nurse- to the ISPA-model. For the nurse model there is no need for a pharmacy or dispensary as medication is prepared for the patient at a central dispensary and merely handed out by the nurse in patient-ready packs. However, the ISPA model requires a dispensary to be registered with the South African Pharmacy Council. We present these costs separately and it is not included in the average cost comparison, as these medicine rooms should arguably be upgraded regardless of level of pharmaceutical care provided given that it is used for medication storage. It is however important to be aware of this potential additional cost. A case study from the district studied was selected and expenditure costs obtained to provide an idea of the approximate costs of an upgrade (Table 5).

**Table 5:** The cost of upgrading a medicine room to a dispensary

**Description**	**Cost (US Dollars)**
The modification and enlarging of medicine room to dispensary	$2 402.18
Security	$1 089.74
Equipment	
Shelving	$1 137.77
Signage	$10.90
Electronic& electrical	$5 463.72
Reference sources	$166.35
Dispensing	$807.25
General	$401.97
Total	$11 479.88

Data sources: Lizette Monteith, Keth'Impilo; Lindsay Wilson, PGWC HIV directorate and Margaret von Zeil, City of Cape Town.

A total cost of US$11 479.88 was spent in the upgrading of the facility. The costs for the upgrade included enlarging of the medicine room, the installation of concrete beams in the room to improve security, installation of a security gate, shelving and the purchasing of a vaccine refrigerator as well as a backup household refrigerator.

### Sensitivity analysis

A sensitivity analysis was conducted to test what the impact on the results would be if one were to vary some of the assumptions. While the baseline represents the current practice at the facilities, another “best practice” scenario was set up whereby the observed assistance of nursing staff in the pharmacist and ISPA models was disregarded and all patients visited the clinic every two months. The ratio of the cost for the ISPA to nurse models was similar for the baseline scenario (see Table 6). A “worst case” scenario was also set up, which involved nurses according to their estimates (at 90% of their productive time) and assumed that patients attended the clinic monthly. For scenario 2, the ISPA model was still less costly than the nurse model.

**Table 6:** Sensitivity analyses of provider costs

**Scenario**	**Models**	**Assumptions**		**Outcomes**	
		**Staff mix**	**Number of patient visits per year**	**Average provider cost/ visit**	**Annual provider cost per patient**
Baseline	Full-time pharmacist		Based on exit interview	US$6.55	US$50.95
	ISPA			US$5.74	US$61.63
	Nurse-driven			US$10.16	US$99.38
1	Full-time pharmacist	One pharmacist and one pharmacists assistant	6	US$4.61	US$27.64
	ISPA	One supervisory pharmacist and one pharmacists assistant		US$4.46	US$26.77
	Nurse-driven	One supervisory pharmacist and one nurse		US$11.11	US$66.65
2	Full-time pharmacist	One pharmacist, one pharmacist’s assistant and maximum nurse time estimate	12	US$8.81	US$105.72
	ISPA	Supervisory pharmacist, pharmacist’s assistants and maximum nurse time estimate		US$8.92	US$107.08
	Nurse-driven	Supervisory pharmacist, pharmacist’s assistants and maximum nurse time estimate		US$18.80	US$225.62

### Patient preferences

The results of the cost analysis suggest that while both of the decentralized approaches, the ISPA- and nurse-driven pharmaceutical care models, significantly decrease direct and indirect costs to patients when accessing treatment, the ISPA model once implemented would also be the least costly to the provider per patient visit (see Table 4). However, only considering the costs does not give us the full picture of the benefits or limitations of the specific models of care.

During the patient exit interviews, respondents were also questioned about whether they would prefer to receive their medication from the nurse or from the pharmacy. The response was most surprising from facilities where the ISPA model had already been implemented. The majority of respondents from these facilities indicated that they would prefer to receive their medication directly from the nurses. Here are some of their responses:

"“I have to walk past people to get the pharmacy and they might recognise me. Also for the time saved” (Respondent, Pharm clinic 2)"

"“The people ask us so many questions that are not pleasant. I don’t find it easy to collect them [her medication] at the pharmacy ‘coz it is like automatic disclosure of my status to everyone” (Respondent, ISPA clinic 1)"

Many of the responses reflected a spatial component to the stigma of HIV, related to being identified when seen by other community members collecting medication (visually known as ART) from the pharmacy. At one of the ISPA model facilities, the identification of people who are HIV positive was exacerbated by the use of a different colored folder for those who are not on ART.

The fear of stigma and the value of anonymity also played a central role in patients’ choice of health facility. As some explained:

"“I feel safer here; the people do not know me here. [It] would’ve been cheaper to go to [facility name] but [I] still rather come here.” (Respondent, Pharm clinic 1)"

"“There are too many people in [facility name] and a lot of people there talk badly about HIV people.”(Respondent, Pharm clinic 1)"

"“My child gets ARV’s from [facility name] but I don’t like it there. There are a lot of people there. If you go that side everyone knows you are positive. Here [current facility] we are not separate.” (Respondent, Nurse clinic 1)"

While respondents preferred receiving their medication directly from the nurse as opposed to the pharmacy as they felt that their anonymity was protected, there was a trade-off. A challenge of the nurse-driven pharmaceutical care model revolved around the logistics of ordering medication for each patient based on their latest prescription in advance of their appointment, and that patients sometimes arrived out of appointment dates, requesting medication. In an attempt to ensure that patient care continues, nurses would then open the pre-packed medication and dispense medication to patients from that source. This is how respondents described it:

"“They give you “bietjie bietjie” [little, little] tablets with other people’s names on. It confuses us.” (Respondent, Nurse clinic 2)"

"“Last month I came and they gave me treatment for another person. Even now they gave me treatment for a week and it’s not in my name.” (Respondent, Nurse clinic 2)"

"“I don’t like the fact that I would come on my date and leave the clinic without getting my pills, I have to take time away from work and my boss is not happy with it.” (Respondent, Nurse clinic 2)"

At one of the facilities, there were also concerns that medication ordered but not collected, was stored in a drawer. This left the stock open to be stolen, and if not stored under the correct conditions, it could compromise patient safety.

## Discussion

Each of the pharmaceutical care models has a unique set of benefits and challenges, as summarized in Table 7. The full-time pharmacist model is presented as the standard of care that would be the ideal at every facility and that has been found to promote rational prescribing and, therefore, been cost saving [51]. However, with the shortage of pharmacists, and an expensive four year training program, this approach would limit the scale-up of ART service provision.

**Table 7:** Comparison of Pharmaceutical Care models

**Pharmaceutical care provider**	**Full-time Pharmacist available**	**Pharmacist’s Assistant (PA) under indirect supervision**	**Nurse-led service provision**
**Overview**	Full-time pharmacist dispensing medication from a prescription written by a doctor, directly to the patient.	The PA works under the indirect supervision of an offsite pharmacist who conducts monthly visits and provides telephonic support. Dispenses directly to patients from a legal prescription written by a doctor. Responsible for stock control. One pharmacist is allowed to supervise up to fivePAs.	This service is often provided in conjunction with an outreach service from a larger centre or in small satellite clinics. Medication is pre-packed by a pharmacist for each patient (patient-ready packs) and delivered to the clinic from which the nurse hands out the medication and monitors the patients’ condition.
**Requirements**	Service is available at larger facilities, for example, at a community health centre. Dispensing is conducted from a pharmacy, operated under the personal supervision of a responsible pharmacist, licensed by the DOH and recorded with SAPC.	Dispensary has to be secure, organized, temperature controlled. Pharmaceuticals and related products are ordered, stored and dispensed directly to clients by the PA and issued to staff for treatment areas. Dispensary design and layout is similar to that of a pharmacy but with smaller floor size.	Storage of medication in a medicine room. The medicine room is intended as a secure, organized, temperature controlled room with limited access, for the bulk storage of pharmaceuticals, for refilling trolleys or cupboards in treatment rooms. No direct patient dispensing, only from patient-ready packs or according to standard operating procedures.
**Benefits**	Highly skilled and trained health professional, experienced in working under pressure and in a team.	More cost-effective in salary and training costs [10]	Increases access to ART for patient
	Promotes rational prescribing and is therefore cost-saving	Onsite to assist in stock management and if patient comes outside of appointment dates.	Patients have established rapport with clinician
		Increases access to ART	There is a perception of time saved if clinician dispenses, though each consultation will take longer.
**Problems**	Scarce	Insufficient training (operational management, dealing with the public/providers)	Prescriber and dispenser is the same person – potential for mistakes.
	Would limit scale-up of ART service	Does not have the authority/skill to promote rational prescribing.	Nurses might not be aware of drug interactions between drug classes.
	Expensive training	Limited pharmacology training	Service is likely to reach saturation point sooner.
	Higher salary level	Lack of career path [52]	Higher salary level than PAs

The benefits of the ISPA model are that PAs undergo only a two year in-service training course supervised by a pharmacist. The PA dispenses directly to the patient and is responsible for stock control. In terms of supervision, one pharmacist can supervise up to five PAs simultaneously. Some concerns are that a PA might not be getting sufficient training, they have limited pharmacology training and do not have the authority to promote rational prescribing. Osman (2005) makes the point that PAs also lack a career path.

The nurse model is very useful in that it provides an option for the rapid scale-up of ART services, although it also reaches the saturation point sooner due to the difficulties of ordering medication for each patient appointment. Medication is not dispensed directly to patients but only from patient-ready packs with the help of standard operating procedures. While there is a perception that waiting time will be reduced when nurses dispense medication, it is more likely that consultation times will be longer and waiting times will increase.

Where there was a lack of doctors to renew prescriptions, this also resulted in increased waiting times and more time spent by nurses being involved in the dispensing process. While nurse prescribing was not a model evaluated in this study, it has been proposed and might conceivably decrease waiting times. However, it does raise concerns when the prescriber and dispenser is the same person, as there is there is a lack of quality control.

The generalizability of this single district evaluation could be seen as a limiting factor given that the functioning of a facility is dependent on many facility-specific characteristics, such as the level of skilled staff, skill mix, the infrastructure available and staff motivation [53]. However, the study does provide a framework within which to evaluate other facilities and some experiences will be common to many facilities. An additional limiting factor to the study is the lack of the facility staff members’ opinions on issues such as the importance of the different color folders and influence on patients’ access to treatment. Further research to explore health professionals’ opinions is recommended.

## Conclusions

In reality, these pharmaceutical care models are not mutually exclusive options and a variety of systems will no doubt be required to achieve scale-up. While the ISPA model is the least costly to the provider and to the patient, the concerns of patients in terms of confidentiality and the avoidance of stigma needs to be addressed as it could negatively impact on patients’ health seeking behaviour. In contrast, the nurse-driven pharmaceutical care model is useful in rapidly scaling-up pharmaceutical care and indeed rolling-out ART service provision to new sites as capital outlay and the recruitment of dispensing personnel is not needed. It does however place a burden on nurses, uses more costly staff and reports suggest that pharmaceutical care may be compromised with patients needing to return to the health facility outside of scheduled appointments to collect medication due to them. Both of these pharmaceutical care models have a place in service provision, but it is imperative to address quality of care and confidentiality concerns of patients.

## Competing interests

The authors declare that they have no conflicts of interest.

## Authors’ contributions

NF and DM conceptualized and designed the study. NF collected the data. NF and DM were involved in the analysis and drafted the manuscript. Both authors read and approved the final manuscript.
